# *In Vivo* Quantitative Ultrasound Image Analysis of Femoral Subchondral Bone in Knee Osteoarthritis

**DOI:** 10.1155/2013/182562

**Published:** 2013-05-27

**Authors:** Jana Podlipská, Juhani M. Koski, Pasi Pulkkinen, Simo Saarakkala

**Affiliations:** ^1^Department of Medical Technology, University of Oulu, Aapistie 7A, P.O. Box 5000, 90014 Oulu, Finland; ^2^Department of Internal Medicine, Mikkeli Central Hospital, 50100 Mikkeli, Finland; ^3^Department of Diagnostic Radiology, Oulu University Hospital, 90029 Oulu, Finland

## Abstract

A potential of quantitative noninvasive knee ultrasonography (US) for detecting changes in femoral subchondral bone related to knee osteoarthritis (OA) was investigated. Thirty-nine patients referred to a knee arthroscopy underwent dynamic noninvasive US examination of the knee joint. The subchondral bone was semiautomatically segmented from representative US images of femoral medial and lateral condyles and intercondylar notch area. Subsequently, the normalized mean gray-level intensity profile, starting from the cartilage-bone interface and extending to the subchondral bone depth of ~1.7 mm, was calculated. The obtained profile was divided into 5 depth levels and the mean of each level, as well as the slope of the profile within the first two levels, was calculated. The US quantitative data were compared with the arthroscopic Noyes' grading and radiographic Kellgren-Lawrence (K-L) grading. Qualitatively, an increase in relative subchondral bone US gray-level values was observed as OA progressed. Statistically significant correlations were observed between normalized US mean intensity or intensity slope especially in subchondral bone depth level 2 and K-L grading (*r* = 0.600, *P* < 0.001; *r* = 0.486, *P* = 0.006, resp.) or femoral arthroscopic scoring (*r* = 0.332, *P* = 0.039; *r* = 0.335, *P* = 0.037, resp.). This novel quantitative noninvasive US analysis technique is promising for detection of femoral subchondral bone changes in knee OA.

## 1. Introduction

Osteoarthritis (OA) is a progressive musculoskeletal disease affecting the entire joint and causing severe disability of the adult population [1, 2]. It accounts for 40% to 60% of all diagnosed degenerative arthritic diseases [3]. OA changes at tissue level are characterized by degeneration and loss of hyaline cartilage, progressive thickening, remodelling and sclerosis of subchondral bone, formation of osteophytes, as well as joint effusion and synovitis [4]. The typical global joint deterioration causes chronic pain and functional impairment leading to loss of working ability, high medical costs, and thereby to negative socioeconomic impact [3, 5]. 

In OA diagnosis, in addition to clinical examination, a conventional radiography (CR) is the most frequently used imaging modality in routine clinical practice [6, 7]. It is able to depict joint space narrowing, marginal osteophytes, subchondral bone sclerosis, cyst and deformity, caused by the disease. However, limitations in CR, such as low sensitivity to very early bone changes, impossibility to directly depict the articular cartilage, synovium, menisci and other soft tissues, as well as inability of three-dimensional joint assessment, make this diagnostic modality insensitive to detection of the initial joint involvement of OA [8, 9].

In the last few years, the role of noninvasive ultrasonography (US) has been highlighted in the OA research [7, 9–12]. US has been reported to be sensitive imaging technique for detection of early and late structure damage of the OA joint [9]. Generally, clinical US is considered as time- and cost-effective, widely available bedside procedure allowing real-time visualization of the joints with minimal patient discomfort and lack of ionizing radiation [7]. In the knee joint noninvasive US is able to depict simultaneously femoral subchondral bone, articular cartilage, and other anatomical structures. It is notable that recent progressive technological development of high-resolution transducers and ultrasound devices enables very detailed musculoskeletal digital imaging [7, 13]. Furthermore, dynamic imaging gives the opportunity of multiplanar investigation of distinct joint areas [7]. Additionally, US offers possibility to monitor the disease progression and follow up the response of different experimental treatments [7]. 

To date, noninvasive US has been concentrated especially on qualitative or semiquantitative grading of OA changes only in articular cartilage [12, 14, 15]. In a recent study, we reported that noninvasive knee US, together with semiquantitative grading of cartilage, has a high positive predictive value for the arthroscopic degenerative changes of the cartilage [12]. However, it is well known that in OA the subchondral bone sclerosis and osteophyte formation appear parallel to cartilage degeneration [2, 16], and it has been even suggested already in the 1986 by Radin and Rose that the initiation of the cartilage degradation can be driven by stiffening of the subchondral bone [17]. Furthermore, also recent findings suggest that subchondral bone may significantly contribute to initiation and/or progression of the disease, and bone alteration may be even preceding the cartilage involvement [2, 18, 19]. Therefore, it is important to focus also on quantification of OA activity and early changes occurring in cartilage-bone interface. 

Quantitative *in vitro *studies have demonstrated that the ultrasound reflection from the cartilage-bone interface is increasing with more severe grade of OA [20–22]. This is caused by the higher acoustic impedance in sclerotic bone since acoustic impedance is directly related to density that increases in sclerosis [20]. However, although all above-mentioned studies approved the ability of the US to depict the degenerative changes of the subchondral bone, there are no studies in which the OA changes would have been quantitatively evaluated from the cartilage-bone interface. We hypothesize that quantitative analysis of US B-mode images could provide more sensitive and specific information about the tissue-level compositional and structural changes in OA.

In this study, a potential of quantitative noninvasive knee US for detecting changes in femoral subchondral bone related to knee OA was investigated. Subchondral bone areas were quantitatively analyzed from US B-mode images and compared with conventional radiography using Kellgren-Lawrence (K-L) grading scale [23], and with arthroscopy using Noyes' grading scale [24].

## 2. Methods

### 2.1. Subjects

In the present study, the acquired US data from our recent study [12] were quantitatively analyzed. The original study involved 40 randomly selected nonrheumatoid patients above the age of 30 years, whereas the current study involved 39 (15 women and 24 men) due to missing representative images of one subject [12]. All of them were referred to a knee arthroscopy because of knee pain. The mean age was 52 years (range 37–73 years) and mean body mass index (BMI) was 27.5 (range 24–35). The informed consent was obtained from all patients and the study was approved by institutional ethics committee.

### 2.2. Ultrasonography

The protocol for knee ultrasonography has been comprehensively described in our recent study [12]. Briefly, before the arthroscopy, all patients underwent noninvasive dynamic knee US examination [12]. The commercially available ultrasound device (Esaote Technos 2000, Esaote Biomedica, Genova, Italy) with 13 MHz linear transducer (LA424) was used in the B-mode imaging of the knee joint. Most of the imaging parameters were kept constant in the US examinations. However, the image depth, focus length, and gain values varied in some patients in order to achieve the best representative images. Some of these changes influenced the gray-level content or resolution of the image and therefore normalization and interpolation of the intensity values were conducted in the quantitative image analysis. 

During the US imaging procedure, the patients were positioned supine with the knee in full flexion (the angle approximately 120°). The probe was placed transversally in the suprapatellar area with the transmitted beam manually kept perpendicular to the femoral surface in order to achieve the finest depiction of femoral condyles and intercondylar notch (later sulcus area). The three main locations, medial (MED) and lateral (LAT) femoral condyles and sulcus area (SULC), were scanned by continuous proximal-distal probe moving (sweeping). The most representative US image of each location, that is, the one which best corresponded visually the overall subjective impression of dynamic imaging [12], was obtained during real-time scanning and saved in DICOM format for later analysis. 

### 2.3. Arthroscopy

At the same day after the US examination, patients underwent the diagnostic arthroscopy of the same knee imaged by US. The surface cartilage abnormalities in femoral MED, LAT, and SULC area were graded by the seven-step Noyes' semiquantitative scoring system [24]: grade 0 is normal; grade 1A represents mild softening or colour changes of the cartilage; grade 1B severe softening or colour changes; grade 2A partial cartilage defect of less than 50%; grade 2B partial cartilage defect more than 50% but less than 100%; grade 3A 100% defect of the cartilage with normal bone; and grade 3B is 100% defect with bone erosion. To simplify the analysis, the grading was converted as follows: grade 0 = 0, grade 1A = 1, grade 1B = 2, grade 2A = 3, grade 2B = 4, grade 3A = 5, and grade 3B = 6.

### 2.4. Radiography

The conventional weight-bearing radiography of the knee joints was conducted for 31 out of 39 patients within 4 months before the US examination and arthroscopy using anteroposterior technique, that is, the knee in extension and weight distributed evenly on both legs. The severity of the OA was determined from radiographs according to the traditional Kellgren-Lawrence scoring system (K-L), ranging from 0 (no OA) to 4 (severe OA) [23]. Radiographs were evaluated by a single observer (JMK). He was blinded to the clinical, sonographic, and arthroscopic data.

### 2.5. Image Analysis

**Figure 1:**
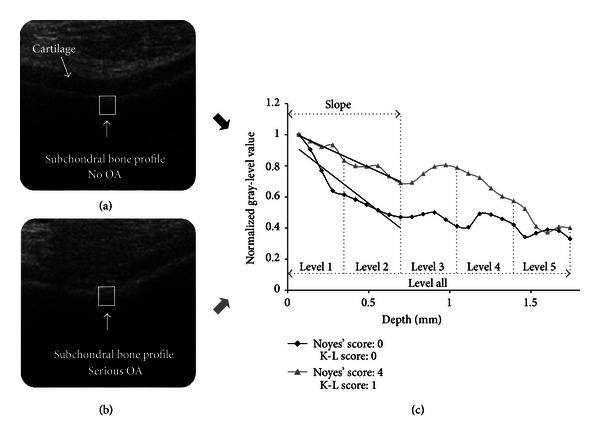
(a) Ultrasound image of healthy knee cartilage-bone interface. A rectangular bone segment was selected in location perpendicular to the incident ultrasound beam. (b) Ultrasound image of osteoarthritic knee cartilage-bone interface. (c) Comparison of nonosteoarthritic (black) and osteoarthritic (gray) subchondral bone gray-level intensity profiles demonstrating decreasing subchondral bone reflection with depth. Five uniform depth levels, overall bone level and slopes calculated for first 2 levels are marked.

A custom-made Matlab script (The MathWorks Inc., Natick, MA, USA) was applied in US image analysis. First, the regions-of-interest (ROI) in femoral MED, SULC, and LAT subchondral bone areas were semiautomatically segmented (Figures 1(a) and 1(b)). The segmentation was initiated by placing manually a border line into the cartilage-bone interface, which was perpendicular to the incident US beam, and thus the ultrasound reflection from subchondral bone was here the strongest. Subsequently, the rectangular ROI was automatically selected. The width of the rectangular ROI was set to 2 mm (~29 pixels for images with image depth 31 mm, and ~22 pixels for images with image depth 41 mm) and the initial height to 8 mm. The bone profile vector of mean gray-level intensity values was obtained by averaging values of each horizontal row in the segmented ROI. In order to compare the intensity values between patients and consequently minimize the effect of possibly varying imaging parameters, the bone profile vector was normalized by dividing all values by the maximum value. Subsequently, the profile vector was cut to start from the maximum value. At this point, the varying pixel size derived from different image depth was corrected by a linear data interpolation. The image depth equal to 31 mm and hence the corresponding pixel size (~0.07 mm) was used as a reference. Furthermore, the final height (i.e., the length of the vector) of the ROI was reduced to 25 pixels (~1.74 mm) and 5 consecutive uniform bone depth levels were defined (Figure 1(c)). The sectioning into 5 levels was conducted in order to investigate which bone location is the most sensitive to ultrasonically determined bone changes during OA. The mean of each level and overall mean of the entire profile (level all) were calculated (Figure 1(c)). Additionally, the intensity slope was calculated within the first two levels (i.e., first 10 pixels, depth: ~0.7 mm) where the most changes were expected because of high ultrasound attenuation in deeper bone locations. Finally, the total femoral bone profile vector (FB), mean values in all depth levels, and intensity slope were calculated for each patient as an average of site-specific data (MED, SULC, and LAT).

### 2.6. Statistical Analysis

The statistical analysis was conducted using SPSS software (ver. 20, SPSS Inc., Chicago, IL, USA). The US image-based normalized mean gray-level intensities (US intensity) of different MED, LAT, and SULC bone depth levels, and intensity slopes were correlated with arthroscopic Noyes' scores, and radiographic K-L scores using Spearman's rank correlation analysis. In order to analyse average femoral bone depth levels and intensity slope, the total femoral arthroscopic score 1 (FAS1) ranging from 0 to 18 was obtained by summing all three site-specific Noyes' scores (i.e., MED, LAT, and SULC). Subsequently, the femoral US data were correlated with FAS1 and K-L score. The 95% confidence intervals (*CI*) for all correlation coefficients were calculated by applying the Fisher's *r* to *Z* transformation as described by Altman and Gardner [25].

Student's *t* tests were conducted for different femoral bone levels and intensity slopes using K-L grouping 0 and 1. In order to conduct the test between different FB levels and Noyes' grading (to have a statistically sufficient number of data in different groups, i.e., >6), the femoral arthroscopic score 2 (FAS2) was established by dividing the FAS1 into groups by ranges as follows: grade 0, 0; grade 1, 1–6; grade 2, 7–12; grade 3, 13–18. Consequently, the relationship between the US femoral bone depth levels and intensity slope using FAS2 grouping 1 and 2 was investigated. In all statistical analysis, the results having *P*  value < 0.05 were considered as significant.

## 3. Results

**Figure 2:**
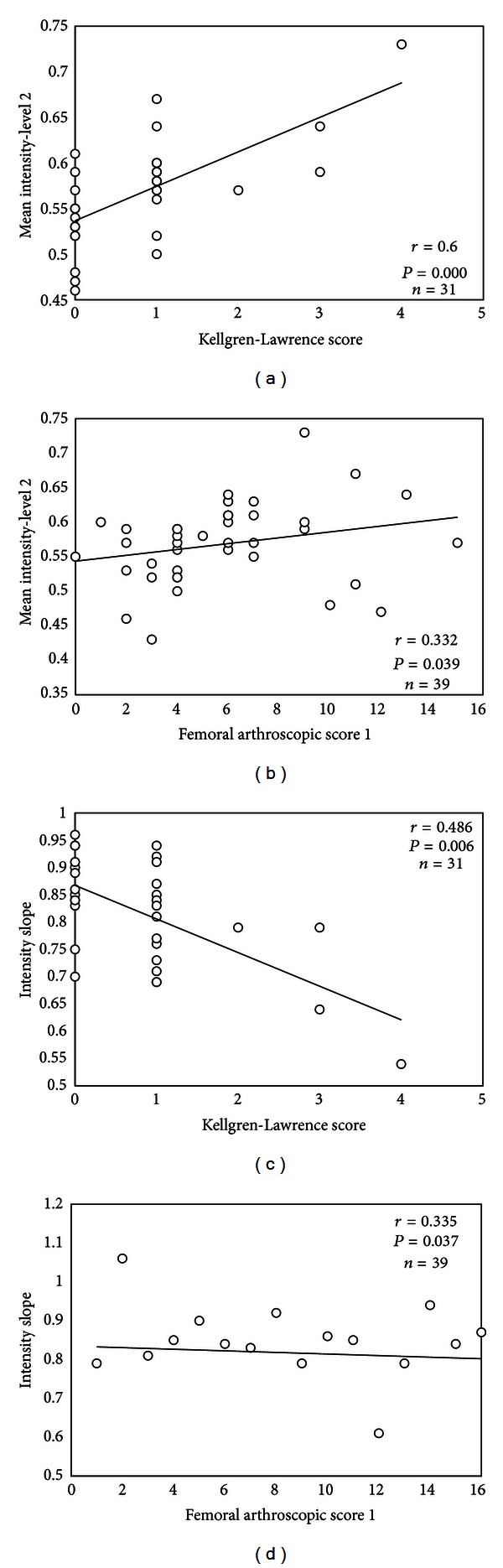
(a) Relationship between normalized mean intensity values of femoral bone level 2 and Kellgren-Lawrence (K-L) grading or femoral arthroscopic score 1 (FAS1) (b). Relationship between femoral subchondral bone intensity slope and K-L grading (c) or FAS1 (d). The slope was calculated from first 2 levels. Please note that the trendline in each plot is only for illustration purposes.

Qualitatively, an increase in normalized subchondral bone US intensity values and decrease in intensity slope were observed as OA progressed (Figure 1(c)). The most distinct intensity variations seemed to appear in subchondral bone depth level 2. Spearman's rank correlations between site-specific bone depth levels 2 and intensity slopes, and K-L and Noyes' grading are presented in Table 1. Statistically significant correlations were found especially between normalized US mean intensity in femoral bone depth level 2 and K-L grading (Figure 2(a)) or FAS1 (Figure 2(b)). The decreasing trend of the absolute femoral bone intensity slope was demonstrated in comparison with radiographic (Figure 2(c)) as well as arthroscopic (Figure 2(d)) findings as OA progressed. Additionally, femoral bone levels 1, 4, and all the levels together yielded moderate or weak correlations with K-L grading (*r* = 0.491, *P* = 0.005; *r* = 0.378, *P* = 0.036; *r* = 0.464, *P* = 0.009, resp.), whereas no other relationship between US femoral variables and FAS1 was observed (Table 2).

In site-specific results, most of the significant US intensity variations were detected in SULC area in comparison with both radiographic and arthroscopic findings, and in MED condyle in correlation with K-L grading, whereas in LAT area there were no significant differences in intensity values and slopes as OA progressed. The detailed results can be found in Table 3.

**Figure 3:**
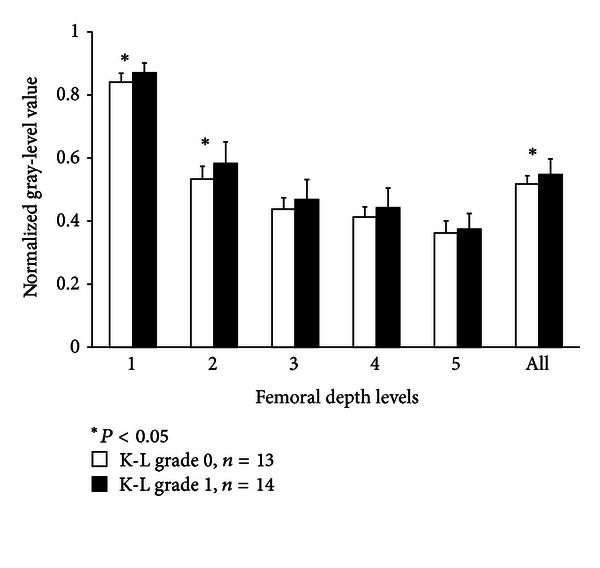
Normalized mean gray-level intensity values in different femoral bone depth levels using grouping radiographic Kellgren-Lawrence (K-L) grade 0 and 1.

**Figure 4:**
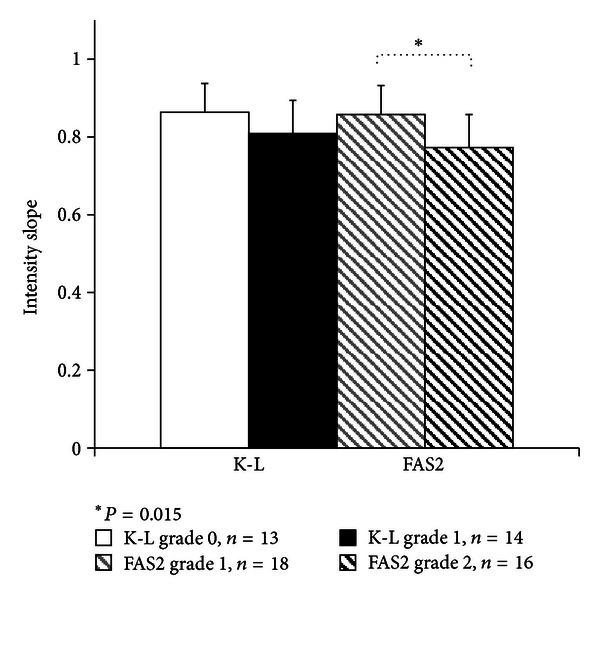
Intensity slopes in different femoral condyles using grouping Kellgren-Lawrence (K-L) grade 0 and 1 and femoral arthroscopic score 2 (FAS2) grade 1 and 2.

Statistically significant increase was observed between normalized US mean intensity values in different femoral bone depth levels and K-L grades 0 and 1 (Figure 3). In comparison of FAS2 grades 1 and 2, the difference was found in bone depth level 2 (*P* = 0.008). The significant decrease was also found between the average femoral intensity slopes of FAS2 groups 1 and 2 (Figure 4).

## 4. Discussion

In this study, the ability of quantitative knee US imaging to detect the knee joint subchondral bone OA changes was investigated *in vivo*. The present intensity profile analysis of subchondral bone from external US images was used for the first time. Current *in vivo* results confirmed the earlier *in vitro* findings [21, 22] that the ultrasound reflection and backscattering from the cartilage-bone interface and subchondral bone increase in OA. This is most probably explained by the bone sclerosis, in which the bone density increases, thus simultaneously increasing the difference in acoustic impedances at the cartilage-bone interface. Our observations support the outcomes of study by Leicht and Raum [20], in which the relationship between articular cartilage and peri-articular bone deterioration was investigated by measuring the acoustic impedance of these tissues. In this study, it was observed that the values in cartilage were higher close to the bone interface and decreased continuously towards the cartilage surface. In subchondral bone, the authors observed significant increase in acoustic impedance closer to the cartilage-bone interface. Therefore, the authors suggested that these findings might indicate the existing subchondral bone sclerosis [20].

The present findings suggest that our quantitative method is able to detect early femoral subchondral bone changes at the cartilage-bone interface, as well as the subchondral bone just beneath. Particularly, the subchondral bone depth level 2, corresponding to actual depth 0.35–0.7 mm inside the subchondral bone, may be considered as the area, where the quantitative changes of US intensity values can be detected most sensitively using this technique. Lower and inconsistent intensity variation in deeper bone levels may be explained by the higher ultrasound attenuation, which decreases the signal-to-noise ratio at these locations [26]. Since there were only few subjects with advanced OA stage (i.e., K-L grade 3 or 4) in this patient group, it is also possible that there were no compositional and structural changes in deeper bone. 

The slope of bone intensity profile was observed to decrease in more severe OA stages. This is likely associated with an increase of subchondral cortical bone volume accounting for pattern of subchondral sclerosis [2, 27]. Another explanation could be disorientation or destruction of integrity of the cartilage-bone interface that can cause US beam scattering and make the US beam reflection appear as a band rather than a line, and thus decrease the slope.

Generally, gray-level intensity US data were more frequently correlating with K-L grading in particular bone sites and different depth levels than with the arthroscopic score. This observation can be explained by the ability of CR to depict subchondral bone. In addition, the evaluation of bone abnormalities is also included in K-L grading [6, 8], whereas in routine arthroscopy, the subchondral bone cannot be assessed before the cartilage is worn off, that is, when Noyes' grade 3A or 3B is diagnosed [24]. However, statistically significant correlations between US analysis of subchondral bone and arthroscopic cartilage scores were still observed, hence confirming the fact that the bone OA changes are occurring parallel with cartilage OA degeneration. 

Most of the US reflection intensity variation was detected in SULC area in both radiographic K-L and arthroscopic site-specific comparisons. Particularly, the correlation coefficients were the highest in bone depth level 2 for both US intensity and slope (Table 1). The main reason for this result may be the best accessibility of femoral SULC area by US. The reflection and backscattering of the bone tissue are highly dependent on and sensitive to the angle of incidence of the US beam [28]. According to our experience, it is easier to meet the condition of perpendicularity of the upcoming beam just in the location of the intercondylar notch area than at the adjacent condyles due to their naturally curved shape. Maintaining a consistent angle of the US beam against the femur might be difficult with manual placement, and thus might affect the US intensity or slope of the cartilage-bone interface. One approach to overcome this problem could be mechanical scanning, as described by Ohashi et al. [29]. In femoral LAT compartment, the statistically insignificant comparisons were probably entailed by a limited acoustic window. In knee flexion, the patella is shifted over the LAT condyle causing acoustic shadow allowing the US beam to reach only small area of the LAT compartment. Therefore, the possible damage visible in radiography or arthroscopy might not have been detected by US. 

The US images were obtained of a previous study protocol and then reviewed for this study what may be considered as a limitation of the present work. Hence, a rather small patient group was enrolled in the study which could explain the relatively large US intensity variation in K-L grade 0 and 1 which can be noticed from Figures 2(a) and 2(c). Additionally, US data in moderate and severe K-L grades were lacking, as well as in completely normal OA (grade 0) and early OA summed arthroscopic grades. Consequently, a comparison of FAS2 groups 0 and 1 was not possible to conduct due to statistically insufficient amount of data in group 0. Therefore, we suggest that more healthy volunteers and symptomatic patients should be enrolled into next studies in which ethically convenient, reliable, quantitative, and noninvasive diagnostic method would be used as a reference (e.g., MRI) in order to verify and validate this method. New low dose, high resolution cone beam computed tomography [30] could be also used as a minimally invasive reference method to quantitative US imaging of subchondral bone and articular cartilage (contrast agent injection needed for visualization of the cartilage).

In the US image analysis, some errors might be caused by the subjective segmentation of ROIs due to possible inclusion of cartilage tissue into the processed ROI. This could happen especially in MED and LAT condyle image segments where the entire bone-cartilage interface was not always totally perpendicular to the upcoming US beam. 

In future studies, the above-mentioned limitations should be taken into account during both, the preparation of image acquisition protocol as well as the image processing and analysis. For instance, the US operator-dependent parameters should be always kept constant in order to compare absolute reflection values within the investigated population. On the other hand, those parameters which can be corrected later during the image analysis, such as image depth, can be set for each patient individually in order to obtain the best representative image. Furthermore, the opportunity of the dynamic imaging should be profited to the overall quantitative assessment of the entire femoral condyle area reachable by noninvasive US, and fully automatic image segmentation of ROIs could be developed in order to make the analysis faster, more precise and accurate. Additionally, more quantitative variables should be introduced and tested, for example, texture features, involving also articular cartilage in order to develop novel OA classification.

The current results are in good line with our previous study using the same patient material, in which we reported that the articular cartilage changes can be also evaluated with noninvasive US by using a semiquantitative cartilage grading scale [12]. Additionally, our study also supports findings published by Saarakkala et al. [21] who studied the degenerative changes in the cartilage-bone interface during locally developed spontaneous cartilage degeneration using the quantitative 2D ultrasound imaging. That study suggested that simultaneous measurements of cartilage surface roughness and ultrasound reflection from the cartilage-bone interface complement each other and thus could be used as more sensitive quantitative diagnostic tool of early OA or followup after surgical cartilage repair [21].

As a conclusion, the significant correlations between femoral US data and radiographic K-L scores, as well as femoral arthroscopic scores, indicate the potential of *in vivo* quantitative US to detect the early knee OA changes at the femoral cartilage-bone interface and subchondral bone. We believe that more sophisticated, combined quantitative analysis of articular cartilage and subchondral bone from US images might provide even more sensitive indicator of early knee OA onset. However, further development of noninvasive quantitative US imaging method and performance of larger trials including symptomatic as well as asymptomatic patient cohort is needed.

## Figures and Tables

**Table 1:** Spearman's rank correlations (*r*) between ultrasound medial (MED), sulcus (SULC), and lateral (LAT) bone depth level 2 intensities and intensity slopes and radiographic K-L grading or arthroscopic Noyes' grading.

	K-L grading			Noyes' grading		
	*n*	*r*	95% CI	*n*	*r*	95% CI
MED level 2	31	0.419*	0.076–0.673	38	0.292	−0.031–0.559
SULC level 2	30	0.541**	0.224–0.754	38	0.372*	0.059–0.618
LAT level 2	31	0.117	−0.248–0.452	38	−0.038	−0.353–0.285
MED slope	31	0.360*	0.006–0.634	38	0.325*	0.006–0.584
SULC slope	30	0.482**	0.147–0.718	38	0.427**	0.124–0.657
LAT slope	31	−0.049	−0.396–0.311	38	−0.095	−0.402–0.232

*P < 0.05.

**P < 0.01.

**Table 2:** Spearman's rank correlations (*r*) between ultrasound femoral (FB) bone depth level intensities and intensity slope and radiographic K-L grading or femoral arthroscopic scoring 1 (FAS1).

	K-L grading			FAS1		
	*n*	*r*	95% CI	*n*	*r*	95% CI
FB level 1	31	0.491**	0.165–0.720	39	0.139	−0.185–0.435
FB level 2	31	0.600***	0.312–0.787	39	0.332*	0.018–0.586
FB level 3	31	0.310	−0.050–0.598	39	0.195	−0.128–0.481
FB level 4	31	0.378*	0.027–0.646	39	−0.016	−0.330–0.301
FB level 5	31	0.238	−0.127–0.547	39	0.053	−0.267–0.362
FB level all	31	0.464**	0.131–0.703	39	0.161	−0.163–0.453
FB slope	31	0.486**	0.159–0.717	39	0.335*	0.022–0.588

*P < 0.05.

**P < 0.01.

***P < 0.001.

**Table 3:** Spearman's rank correlations (*r*) between ultrasound medial (MED), sulcus (SULC), and lateral (LAT) bone depth level intensities and intensity slopes and radiographic K-L grading or arthroscopic Noyes' grading.

	K-L grading			Noyes' grading		
	*n*	*r*	95% CI	*n*	*r*	95% CI
MED level 1	31	0.457**	0.122–0.698	38	0.162	−0.166–0.458
MED level 2	31	0.419*	0.076–0.673	38	0.292	−0.031–0.559
MED level 3	31	0.373*	0.021–0.642	38	0.192	−0.136–0.482
MED level 4	31	0.405*	0.059–0.664	38	0.133	−0.195–0.434
MED level 5	31	0.232	−0.133–0.542	38	0.115	−0.213–0.419
MED level all	31	0.418*	0.075–0.673	38	0.167	−0.161–0.462
MED slope	31	0.360*	0.006–0.634	38	0.325*	0.006–0.584
SULC level 1	30	0.244	−0.128–0.555	38	−0.022	−0.339–0.300
SULC level 2	30	0.541**	0.224–0.754	38	0.372*	0.059–0.618
SULC level 3	30	0.390*	0.035–0.658	38	0.462**	0.167–0.681
SULC level 4	30	0.202	−0.171–0.524	38	0.242	−0.084–0.521
SULC level 5	30	0.201	−0.172–0.523	38	0.282	−0.041–0.552
SULC level all	30	0.394*	0.039–0.661	38	0.358*	0.043–0.608
SULC slope	30	0.482**	0.147–0.718	38	0.427**	0.124–0.657
LAT level 1	31	0.109	−0.255–0.446	38	0.029	−0.293–0.345
LAT level 2	31	0.117	−0.248–0.452	38	−0.038	−0.353–0.285
LAT level 3	31	−0.179	−0.501–0.187	38	−0.091	−0.399–0.236
LAT level 4	31	−0.080	−0.422–0.282	38	0.063	−0.262–0.375
LAT level 5	31	−0.009	−0.362–0.347	38	0.093	−0.234–0.401
LAT level all	31	0.062	−0299–0.407	38	0.008	−0.312–0.327
LAT slope	31	−0.049	−0.396–0.311	38	−0.095	−0.402–0.232

*P < 0.05.

**P < 0.01.
