# Corrigendum to “*In Vivo* Quantitative Ultrasound Image Analysis of Femoral Subchondral Bone in Knee Osteoarthritis”

**DOI:** 10.1155/2016/5950841

**Published:** 2016-09-14

**Authors:** Jana Podlipská, Juhani M. Koski, Pasi Pulkkinen, Simo Saarakkala

**Affiliations:** ^1^Research Unit of Medical Imaging, Physics and Technology, University of Oulu, Aapistie 7A, POB 5000, 90014 Oulu, Finland; ^2^Department of Internal Medicine, Mikkeli Central Hospital, 50100 Mikkeli, Finland; ^3^Department of Diagnostic Radiology, Oulu University Hospital, 90029 Oulu, Finland; ^4^Medical Research Center, University of Oulu and Oulu University Hospital, 90014 Oulu, Finland

 In the article titled “*In vivo* quantitative ultrasound image analysis of femoral subchondral bone in knee osteoarthritis” [1], there was an error in the data set (scatter plot) of provided Figure 2(d). The correlation, however, was correct and consequently it does not change our conclusions. Here we provide Figure 2(d) with the corrected data set. Additionally, in Figure 2(a) the mathematical expression of *P* value was corrected from “*P* = 0.000” to more appropriate “*P* < 0.001.”

## Figures and Tables

**Figure 2:**
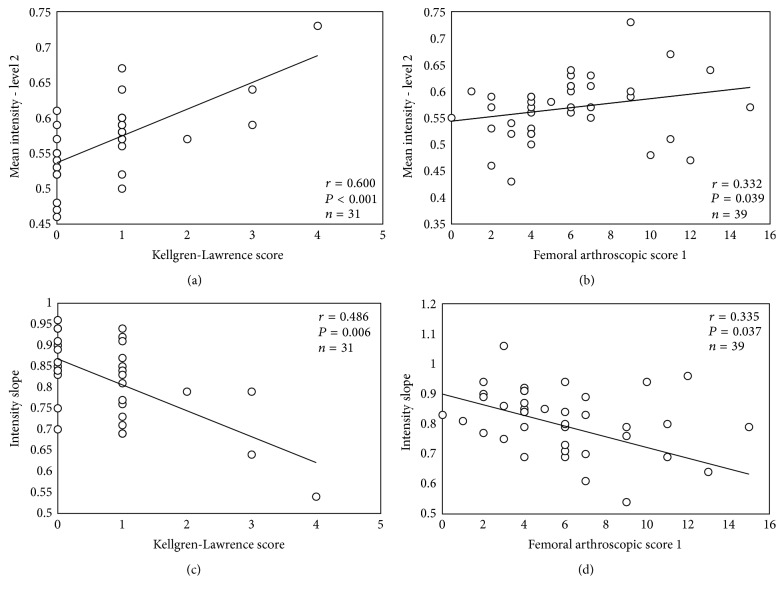
(a) Relationship between normalized mean intensity values of femoral bone level 2 and Kellgren-Lawrence (K-L) grading or femoral arthroscopic score 1 (FAS1) (b). Relationship between femoral subchondral bone intensity slope and K-L grading (c) or FAS1 (d). The slope was calculated from the first 2 levels. Please note that the trendline in each plot is only for illustration purposes.
